# Probiotic Supplementation Attenuates Chemotherapy-Induced Intestinal Mucositis in an Experimental Colorectal Cancer Liver Metastasis Rat Model

**DOI:** 10.3390/nu15051117

**Published:** 2023-02-23

**Authors:** Matas Jakubauskas, Lina Jakubauskiene, Bettina Leber, Angela Horvath, Kestutis Strupas, Philipp Stiegler, Peter Schemmer

**Affiliations:** 1 General, Visceral and Transplant Surgery, Department of Surgery, Medical University of Graz, Auenbruggerplatz 2, 8036 Graz, Austria; 2 Faculty of Medicine, Vilnius University, M. K. Ciurlionio str. 21, 03101 Vilnius, Lithuania; 3 Division of Gastroenterology and Hepatology, Medical University of Graz, Auenbruggerplatz 2, 8036 Graz, Austria

**Keywords:** probiotics, chemotherapy, FOLFOX, mucositis, colorectal cancer, liver metastasis

## Abstract

The use of chemotherapeutic agents is of paramount importance when treating colorectal cancer (CRC). Unfortunately, one of the most frequent chemotherapy (CTx) side effects is intestinal mucositis (IM), which may present with several clinical symptoms such as nausea, bloating, vomiting, pain, and diarrhea and even can result in life-threatening complications. There is a focused scientific effort towards developing new therapies to prevent and treat IM. The aim of this study was to assess the outcomes of probiotic supplementation on CTx-induced IM in a CRC liver metastasis rat model. Six-week-old male Wistar rats received either a multispecies probiotic or placebo mixture. On the 28th experiment day, rats received FOLFOX CTx, and afterwards, the severity of diarrhea was evaluated twice daily. Stool samples were collected for further microbiome analysis. Additionally, immunohistochemical stainings of ileum and colon samples with were performed with MPO, Ki67, and Caspase-3 antibodies. Probiotic supplementation alleviates the severity and length of CTx-induced diarrhea. Additionally, probiotics significantly reduced FOLFOX-induced weight and blood albumin loss. Furthermore, probiotic supplementation mitigated CTx-induced histological changes in the gut and promoted intestinal cell regeneration. This study shows that multispecies probiotic supplementation attenuates FOLFOX-induced IM symptoms by inhibiting apoptosis and promoting intestinal cell proliferation.

## 1. Introduction

Colorectal cancer (CRC) is a major oncologic burden responsible for around 10% of new cancer cases and deaths worldwide [1]. As in many types of cancers, the use of chemotherapeutic agents is of paramount importance when treating CRC [2]. Unfortunately, one of the most frequent chemotherapy (CTx) side effects is intestinal mucositis (IM). It can affect 40 to 100% of cancer patients depending on the drug and its dosing [3,4]. IM develops due to the direct cytotoxicity inflicted by the antineoplastic drugs on the intestine epithelial cells, and this further promotes inflammation and indirect injury such as villus blunting or loss of the mucus layer [5,6]. This intestinal injury may present with various clinical symptoms such as nausea, vomiting, bloating, pain, and diarrhea and even can lead to life-threatening complications [4,7]. Furthermore, severe IM may result in suboptimal cancer treatment as the CTx doses need to be reduced or postponed.

There is a focused scientific effort towards developing new therapies to prevent and treat IM [6]. One of the main IM pathophysiological mechanisms is the gut microflora balance alteration; thus, probiotics and antibiotics are being tested to prevent the formation of a harmful environment within the intestine [8,9]. Probiotics are described as “non-pathogenic live microorganisms that, when administered in adequate amounts, confer a health benefit on the host” [10]. They have a very wide variety of actions on the host, such as direct interaction with pathogens, promotion of intestinal barrier function, or immunomodulation [11]. As pointed out by a recent review, several studies report positive results of different probiotic strains in treating IM; however, further research for novel probiotic strains and their action mechanisms is essential [8].

The aim of this study was to assess the outcomes of a novel multispecies probiotic combination on CTx-induced IM in a CRC liver metastasis rat model.

## 2. Materials and Methods

### 2.1. Animals

In this experimental model, we used six-week-old Wistar rats (Janvier Labs, Le Genest-Saint-Isle, France) ranging in weight from 200 to 280 g at the start of the protocol. According to the experimental groups, rats were housed two to four per enclosure, having ad libitum pelleted chow and tap water. The experimental protocol was approved by the Austrian Committee for Animal Trials (Approval number: BMWF-66.010/0158-V/3b/2019) and performed according to the 3R guidelines.

### 2.2. Experiment Design

**Figure 1:**
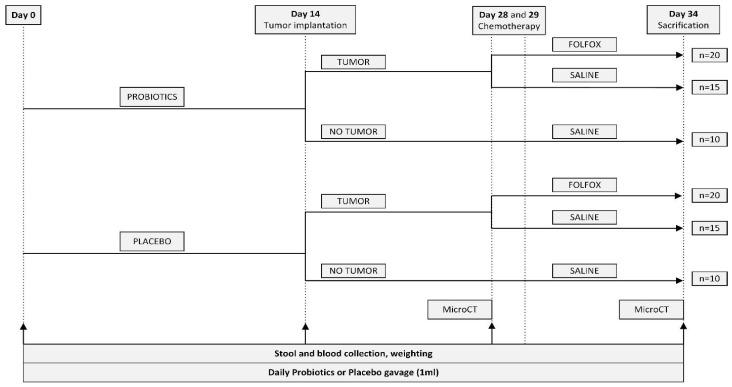
Study design.

Overall, 90 rats were separated into six different study groups (Table 1). The detailed study design is presented in Figure 1. The whole study protocol lasted for 34 days. For the first two weeks, rats received daily gavage with probiotics or placebo mixture. On day 14, rats underwent CRC liver metastasis implantation that used a rat colorectal cancer cell line (CC531) or sham surgery. On days 28 and 29, CTx drugs or placebo were administered. After administering the first dose of CTx, diarrhea assessment started. On day 34, after performing necessary imaging, modalities rats were culled, and samples were collected.

### 2.3. Probiotics

A multispecies probiotic mixture (provided by Institut Allergosan, Graz, Austria) composed of eight bacterial strains was used. *Lactobacillus casei W56; Lactobacillus acidophilus W37; Lactobacillus brevis W63; Lactococcus lactis W58; Bifidobacterium lactis W52; Lactococcus lactis W19; Lactobacillus salivarius W24; and Bifidobacterium bifidum W23* were combined with 1 g of matrix (maize starch, maltodextrins, vegetable protein, potassium chloride, magnesium sulphate, amylases, and manganese sulphate). The placebo contained only the matrix. Either the probiotic or placebo powder was dissolved freshly every morning using tap water approximately 15 min before gavaging. Each rat received 1 mL of probiotic (1.2 × 10^9^ CFU/mL) or placebo suspension.

### 2.4. Chemotherapy

**Figure 2:**
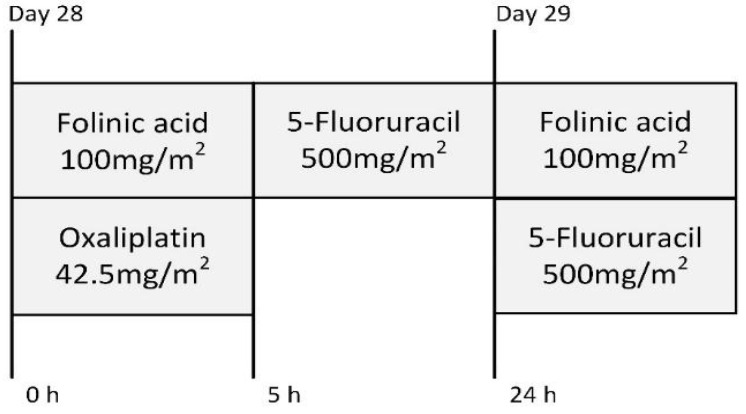
FOLFOX CTx dosage scheme.

In this study, we used the FOLFOX regimen, as it is used for colorectal cancer metastasis treatment and has a proven capability to induce gastrointestinal damage [12,13,14]. The doses were adjusted using a previously described methodology according to the animals’ skin surfaces [15]. All CTx drugs were injected intraperitoneally under general 2% isoflurane anesthesia. The CTx administration protocol is presented in Figure 2.

### 2.5. Diarrhea Evaluation

**Figure 3:**
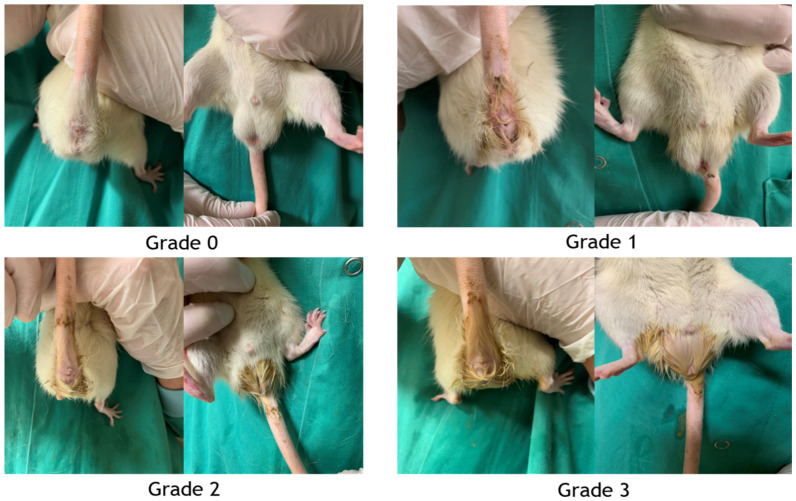
Diarrhea assessment. Grade 0, no diarrhea; grade 1, mild diarrhea (staining of anus); grade 2, moderate diarrhea (staining of the lower abdomen); grade 3, severe diarrhea (staining over legs and higher abdomen or continual oozing).

All animals were examined twice daily after the first injection of the chemotherapeutical agents. Diarrhea was graded using a published scale: grade 0, no diarrhea; grade 1, mild diarrhea (staining of anus); grade 2, moderate diarrhea (staining of the lower abdomen) and; grade 3, severe diarrhea (staining over legs and higher abdomen or continual oozing) (Figure 3) [16].

### 2.6. Blood Tests

The first three samples were acquired by drawing blood from the subclavian vein. The last blood sample was drawn on day 34 from the inferior vena cava just before final organ sample collection. Complete blood count was calculated using a V-Sight hematology analyzer (A. Menarini Pharma GmbH, Vienna, Austria). Biochemical blood measurements were performed with a Spotchem EZ (A. Menarini Pharma GmbH, Vienna, Austria) analyzer.

### 2.7. Immunohistochemical Staining

Organ samples were fixed in 4% buffered formaldehyde solution, rinsed with distilled water, and dehydrated with ascending ethanol series. After incubating at 60 °C, samples were embedded in paraffin. Using a rotary microtome, 2 μm thick tissue sections were cut.

Slides were stained using the following primary antibodies: Anti-MPO (Dako, Via Real Carpinteria, CA, USA, A0398, dilution 1:800), Anti-Ki67 (Abcam, Cambridge, UK; ab16777, dilution 1:200), and Anti-Caspase-3 (Abcam, Cambridge, UK; ab4051, dilution 1:200). The UltraVision LP Detection System: HRP Polymer (Thermo Fisher Scientific, Waltham, MA, USA) and DAB Chromogen (Dako, Via Real Carpinteria, CA, USA) were used to visualize the target antigen. Sections were counterstained with hematoxylin.

All stained slides were scanned and analyzed using the open-source QuPath software (v0.3.0) [17].

### 2.8. Intestine Crypt and Villi Length Analysis

Crypt and villi length in both ileum and colon were measured according to a publication by Adelman et al. [18]. Five random crypt and villi lengths of the ileum were measured, and a villi/crypt length ratio was calculated for further data analysis. The median value of five random crypt lengths of the colon for each rat was used.

### 2.9. Microbiome Analysis

DNA isolation from fecal samples was performed using the Magna Pure LC DNA III Isolation Kit (Bacteria, Fungi) (Roche, Mannheim, Germany) according to previously reported protocols [19,20]. A stool pellet was mixed with 500 µL phosphate-buffered saline (PBS) and 250 µL bacterial lysis buffer. Afterward, the sample was homogenized using the MagNA Lyser instrument (Roche Life Science. Mannheim, Germany) at 6500 rpm for two 30 s cycles. Enzymatic lysis was performed using 25 µL lysozyme (100 ng/mL, 37 °C for 30 min) and 43.4 µL proteinase K (20 mg/mL, 65 °C for 1 h). After the samples were heat inactivated at 95 °C for 10 min, DNA was extracted using a MagnaPure LC instrument (Roche, Mannheim, Germany) according to the manufacturer’s instructions. Extracted DNA was eluted in 100 μL elution buffer and stored at −20 °C until analysis. Then, 2 μL of total DNA was used in a 25 μL PCR reaction in triplicates using a FastStart High Fidelity PCR system (Sigma, Darmstadt, Germany) according to the manufacturer’s instructions and the target specific primers 515F (5′-GTGYCAGCMGCCGCGGTAA-3′) and 806R (5′GGACTACNVGGGTWTCTAAT-3′) for 30 cycles. Triplicates were pooled, normalized, indexed, and purified according to a published protocol [19]. The final pool was sequenced on an Illumina MiSeq desktop sequencer at 9 pM and v 3 600 cycles chemistry. FASTQ raw reads were processed using QIIME2 tools implemented on a local galaxy instance (https://galaxy.medunigraz.at). Taxonomic assignment was carried out using a naïve Bayesian classifier trained on the SILVA V132 database. Features were summarized on genus level for further analysis. Using the web-based analysis platform “Calypso”, group-specific general linear models identified genera that significantly changed during the course of the study. For the selected genera, differences between day 28 and day 34 were calculated and entered into a Spearman correlation analysis with diarrhea characteristics and albumin and weight changes to assess the associations between microbiome changes and the effects of the probiotics. *p*-values were adjusted with Benjamini–Hochberg correction and visualized using the R package “corrplot” (Version 0.92).

### 2.10. Statistical Analysis

Statistical analysis was executed using SPSS 23.0 (IBM Corp., Armonk, New York, NY, USA) and GraphPad Prism 9 (GraphPad Software, La Jolla, CA, USA). Data distribution was evaluated with the Shapiro–Wilk test. Normally distributed data were further analyzed using *t*-test and one-way ANOVA with Tukey’s post hoc test. Non normally distributed data were investigated with Mann–Whitney U and Kruskal–Wallis with Dunn’s post hoc tests. A *p*-value of 0.05 or lower was considered significant. Data is provided as median and quartiles (Q1; Q3).

## 3. Results

### 3.1. Response to Chemotherapy

During the whole study, we had a single death due to CTx toxicity (Table 1). The administration of FOLFOX CTx induced severe leukopenia for both CTx-receiving rat groups. No probiotic influence was observed on the severity of FOLFOX-induced leukopenia.

### 3.2. Diarrhea Assessment

**Figure 4:**
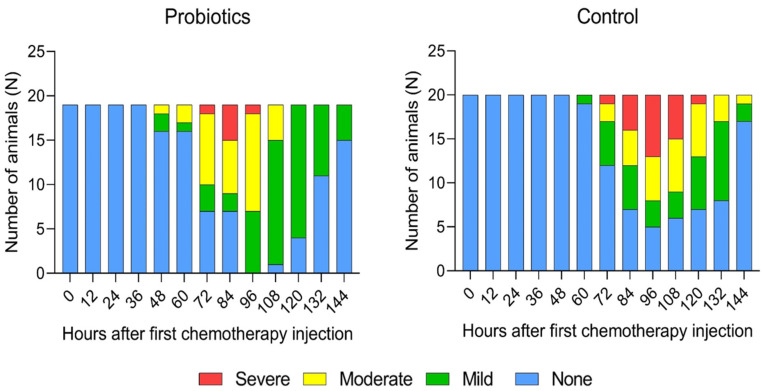
Diarrhea assessment results.

A total of 15 animals (75%) receiving placebo and 19 animals (100%) receiving probiotics developed some degree of diarrhea (Figure 4). The peak incidence of diarrhea was observed 96 h after the first CTx injection in both groups. At the peak incidence, seven animals (35%) receiving placebo and four (21%) receiving probiotics developed severe diarrhea. Furthermore, severe diarrhea tended to resolve quicker for animals receiving probiotic supplementation (24 h (12.0; 30.0) vs. 12 h (12.0; 12.0); *p* = 0.026).

### 3.3. Weight Change

**Figure 5:**
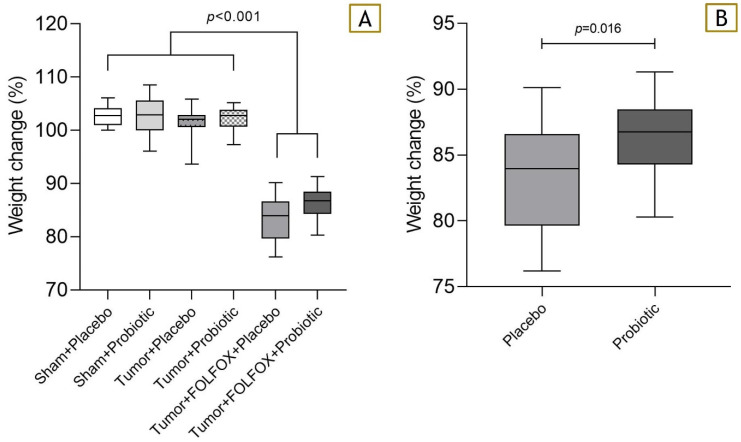
(**A**) Weight change between rats that received FOLFOX CTx and did not. (**B**) Weight change after FOLFOX CTx between probiotic and placebo groups. Weight change calculated as weight day 28 (g) × 100%/weight at day 34 (g).

At the start of the study, rat weight increased evenly in all groups. This tendency continued for rats not receiving CTx, yet rats that received FOLFOX significantly lost weight (Figure 5A). Probiotic supplementation managed to significantly limit weight loss caused by CTx (83.97% (79.65; 86.61) vs. 86.76% (84.29; 88.46); *p* = 0.016) (Figure 5B).

### 3.4. Blood Albumin Levels

**Figure 6:**
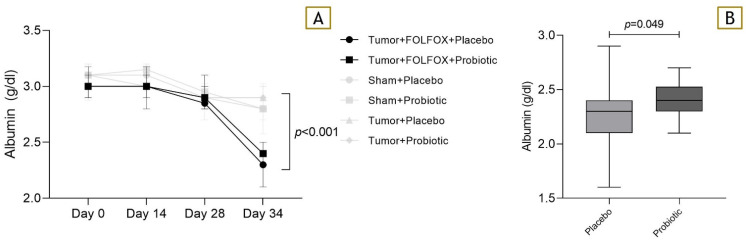
(**A**) Albumin concentration change throughout the study. (**B**) Blood albumin level change in percent between days 28 and 34 after FOLFOX CTx.

Rat blood albumin levels were consistent across all groups at the start of the study (Figure 6A). FOLFOX CTx significantly reduced blood albumin levels in both the placebo and probiotic groups. We further analyzed blood albumin level changes between protocol days 28 and 34. Figure 6B shows that probiotic supplementation managed to significantly reduce CTx-induced blood albumin loss (80.35% (68.03; 84.88) vs. 83.60% (80.28; 89.48); *p* = 0.021).

### 3.5. Histopathological Examination

**Figure 7:**
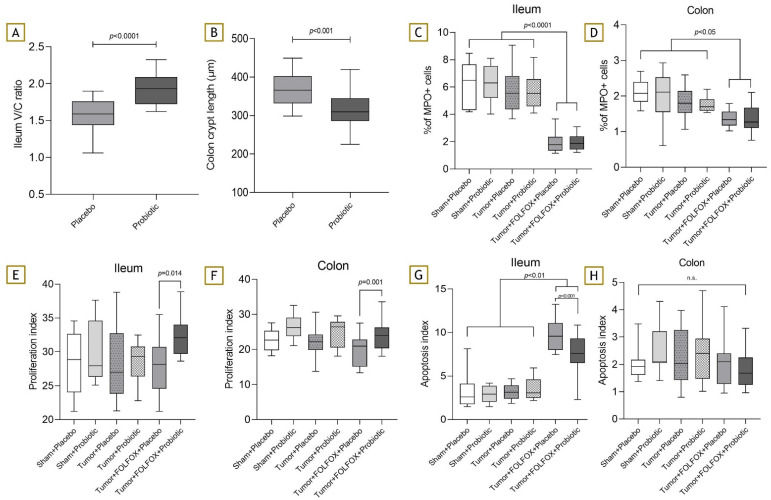
Ileum and colon immunohistological analysis. (**A**) Terminal ileum villi/crypt length ratio after FOLFOX CTx. (**B**) Colon crypt length after FOLFOX CTx. (**C**) MPO+ cells in the terminal ileum. (**D**) MPO+ cells in the colon. (**E**) Proliferation index in the terminal ileum; (**F**) proliferation index in the colon. (**G**) Apoptosis index in the terminal ileum; (**H**) apoptosis index in the colon. n.s.—not significant.

Analysis of rat terminal ileum villi length and crypt depth ratio showed that probiotics helped to alleviate CTx-induced intestinal damage (1.59 (1.44; 1.76) vs. 1.93 (1.73; 2.09); *p* < 0.001)) (Figure 7A). A similar tendency was seen with the rat colon crypt depth analysis (366.00 µm (331.70; 402.20) vs. 309.80 µm (286.20; 345.10; *p* < 0.001)) (Figure 7B). The percentage of MPO-positive cells was significantly lower in both FOLFOX-receiving groups in comparison to rats that did not receive CTx, and this was observed both in colon and ileum tissues. There were no differences between both CTx groups (Figure 7C,D). Probiotic supplementation greatly increased intestinal cell proliferation, and the differences between both CTx groups were statistically significant (Figure 7E,F). Furthermore, it seems that probiotic supplementation also managed to have a protective effect from CTx-induced apoptosis on ileum cells (9.57% (7.98; 11.07) vs. 7.58% (6.50; 9.30); *p* = 0.001) (Figure 7G). However, the apoptosis index in the colon samples was similar across all experiment groups (Figure 7H).

### 3.6. Associations between Adverse Effects and the Microbiome

**Figure 8:**
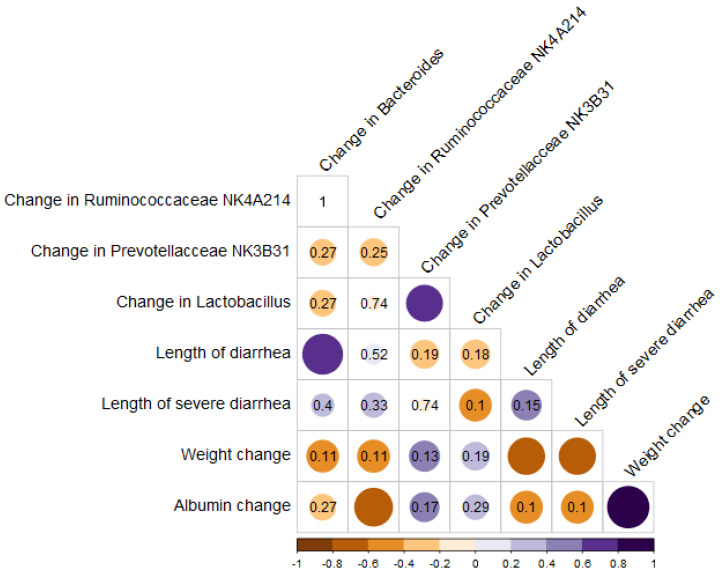
Correlation plot of changes in abundance of bacteria genera and clinical outcomes. r_s_—correlation coefficient; *p*_adj_—adjusted *p*-value.

Correlation analysis results are summarized and presented in Figure 8. The length of diarrhea shows a strong correlation with an increase in *Bacteroides* in stool samples during CTx (r_s_ = 0.76; *p*_adj_ = 0.002). Additionally, our correlation analysis shows that the higher abundance of *Ruminococcaceae NK4A214*-group bacteria may further promote albumin loss during CTx (r_s_ = −0.68; *p*_adj_ = 0.015). Further microbiome representation data are presented in the Supplementary File.

## 4. Discussion

One of the most common CTx side effects is IM, and its occurrence can affect 40 to 100% of cancer patients depending on the CTx regimen used [3,4]. IM interferes with optimal cancer treatment as the CTx doses need to be reduced or postponed; furthermore, it may even cause life-threatening complications [4,7]. Pathophysiology of IM is very complex but mainly involves five phases: (1) direct DNA damage and tissue cytotoxicity; (2) primary damage response, leading to inflammation and apoptosis; (3) signal amplification, resulting in exacerbated tissue injury; (4) inflammation and ulceration, leading to villus atrophy and barrier disruption; and (5) healing, with epithelial proliferation and intestine barrier regeneration [5,6,21]. Although this is a great IM development summary, various CTx regimes act differently on the gut barrier. Currently, only few studies report the impact of FOLFOX CTx on the development of IM and subsequent diarrhea [13,22,23]. Therefore, the aim of this study was to assess the outcomes of probiotic supplementation on FOLFOX CTx-induced IM.

Our study shows that a previously never-tested multispecies probiotic combination alleviates the severity and length of CTx-induced diarrhea. Additionally, probiotics significantly reduced FOLFOX-induced weight and blood albumin loss. Furthermore, probiotic supplementation mitigated CTx-induced histological changes in the gut and promoted intestinal cell regeneration. Lastly, we managed to identify few bacteria groups that may play a role in the pathogenesis of severe diarrhea development and CTx-induced blood albumin level loss.

Similarly, FOLFOX CTx-induced diarrhea attenuation results were reported by Chang et al. [22]. After administering increasing doses of *Lactobacillus casei* variety *rhamnosus*, authors observed lower diarrhea severity scores, with the peak being reached 6 days after the first chemotherapeutical agent injection. The peak diarrhea incidence differs slightly from the one we report in our study (96 h), but this may be mainly explained by the different FOLFOX CTx injection timing and dosage.

One of the most common CTx side effects is weight loss, and on some occasions, it can be associated with worse patient survival [24]. Similarly, to our study, several other studies showed that probiotics containing *Lactobacillus* and *Bifidobacterium* manage to decrease CTx-induced weight loss [25,26]. Additionally, a study by Bowen et al. reported that multispecies probiotic VSL#3 can prevent CTx-caused weight loss. Probiotics preserve weight by preserving the intestinal integrity and alleviating CTx-induced diarrhea [27]. Additionally, the development of hypoalbuminemia has been associated with increased rates of chemotherapy failure and mortality [28,29]. To our knowledge, our study is the first to show that multispecies probiotic supplementation can help to preserve albumin levels in a cancer model.

Probiotic safety and their interaction with the CTx itself for immunocompromised cancer patients is a very important and often debated issue [30,31,32]. In this study, we did not observe an increase in severe complications or premature deaths attributed to probiotic use. Moreover, our other study analyzing the impact of probiotics on tumor growth revealed that the used probiotic supplementation does not decrease the efficiency of FOLFOX CTx on CRC liver metastasis [33].

The histopathological investigation of ileum and colon tissue sheds some light on the underlying probiotic action mechanisms. Probiotic supplementation managed to alleviate CTx damage to the intestine, and this was indicated by the preserved ileum villi/crypt length ratio and crypt depth in the colon. This finding was in line with the results reported by Chang et al. in a CRC model; however, other studies report inconsistent results [22,25,34]. The analysis of anti-MPO-positive cells revealed an unexpected finding. The percentage of anti-MPO-positive cells was significantly lower in both chemotherapy groups both in the ileum and colon. This result is a bit counterintuitive, as usually, various inflammatory cells play an important role in the development of IM [6]. This result may be mostly influenced by the fact that the samples were gathered 6 days after initial CTx administration, when the course of IM starts to shift towards regeneration, especially in rats [5]. Furthermore, rats were in severe leukopenia at that time, theoretically leaving fewer neutrophils for tissue infiltration. A critical event for IM development is increased intestinal cell apoptosis, which was measured using Caspase-3 staining [5]. Interestingly, our results show that probiotics managed to decrease apoptosis in the ileum; however, no positive effects were observed in the colon. This may be mostly explained by the 10-times-lower large intestine apoptotic activity and thus its lower susceptibility to CTx-induced damage [35]. Moreover, probiotic supplementation significantly increased intestinal cell regeneration in both the colon and ileum. Various other probiotic strains have previously shown intestinal-healing effects in CTx-induced IM models [22,27].

Administration of CTx is known to dramatically alter the gut microbiome [36]. This includes the overall decrease in diversity and a relative increase of proteobacteria [37,38,39]. Our performed microbiome correlation analysis revealed that a relative increase of *Bacteroides* group bacteria was associated with increased diarrhea length. Results in the literature are quite inconsistent, as some studies report an increase and some a decrease of *Bacteroides* abundance when administering CTx [37,38]. Furthermore, although *Ruminococcaceae NK4A214*-group bacteria are generally known for their short-chain fatty acid production and anti-inflammatory effects, our analysis indicated contradictive results, showing that the higher abundance of *Ruminococcaceae NK4A214*-group bacteria may further promote albumin loss during CTx [40,41,42]. We could not identify other articles supporting this result; thus, it should be used cautiously and re-evaluated in a further study.

One potential drawback of this study is the adoption of an animal model. Although successful probiotic effect translation from rodent to human has been published, we should note that the multispecies probiotics used in our study may have a different interaction with the human gut microbiome, resulting in altered outcomes [43].

## 5. Conclusions

Our study indicates that multispecies probiotic supplementation attenuates FOLFOX-induced IM symptoms in an experimental rat colorectal cancer liver metastasis model. As shown by the immunohistochemical analysis, the used probiotics act by inhibiting apoptosis and promoting intestinal cell proliferation. Further research into more in-depth molecular mechanisms is warranted, and our study group is conducting an experimental study that will focus more on these multispecies-probiotic-induced gut permeability changes.

## Figures and Tables

**Table 1:** Study groups.

	CONTROL Tumor (−)/CTx (−)		Non FOLFOX Tumor (+)/CTx (−)		FOLFOX Tumor (+)/CTx (+)	
Gavage	Placebo	Probiotics	Placebo	Probiotics	Placebo	Probiotics
Start (n)	10	10	15	15	20	20
End (n)	10	10	15	15	20	19 *

* Premature death on day 34 due to CTx complications. CTx, chemotherapy.

## Data Availability

All data relevant to the study are included in the article.

## Supplementary Materials

The following supporting information can be downloaded at: https://www.mdpi.com/article/10.3390/nu15051117/s1, Figure S1: Normalized alpha diversity changes throughout the study. Figure S2: Alpha diversity panel. Calculated from non-normalized data. Figure S3: Principle coordinate analysis (PCoA) using Bray-Curtis dissimilarity. Figure S4: Phylum level microbiome composition. Figure S5: Class level microbiome composition. Figure S6: Order level microbiome composition. Figure S7: Family level microbiome composition. Figure S8: Genus level microbiome composition.
