# Supplementary material for: Probiotic Supplementation Attenuates Chemotherapy-Induced Intestinal Mucositis in an Experimental Colorectal Cancer Liver Metastasis Rat Model
Source: Nutrients. 2023 Feb 23;15(5):1117. doi: 10.3390/nu15051117 (PMC10005486; doi:10.3390/nu15051117)
Supplement: Supplementary file 1 [file nutrients-15-01117-s001.zip › nutrients-2191198-supplementary.pdf]

Supplementary material

Study time periods:

- S0 - Baseline
- S1 – Before tumor implantation
- S2 – Before receiving chemotherapy
- S3 – End of the protocol

Alpha diversity

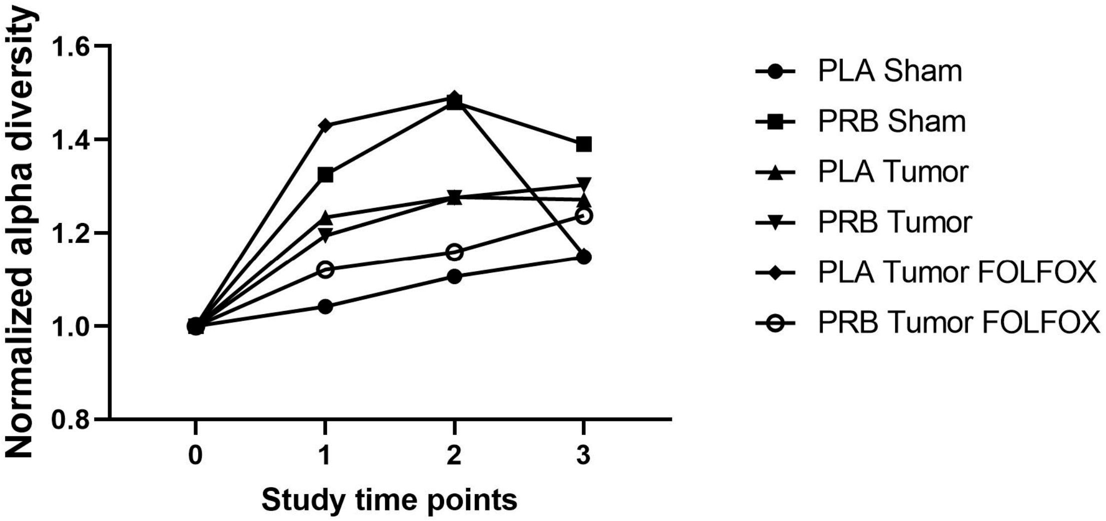

Figure S1. Normalized alpha diversity changes throughout the study

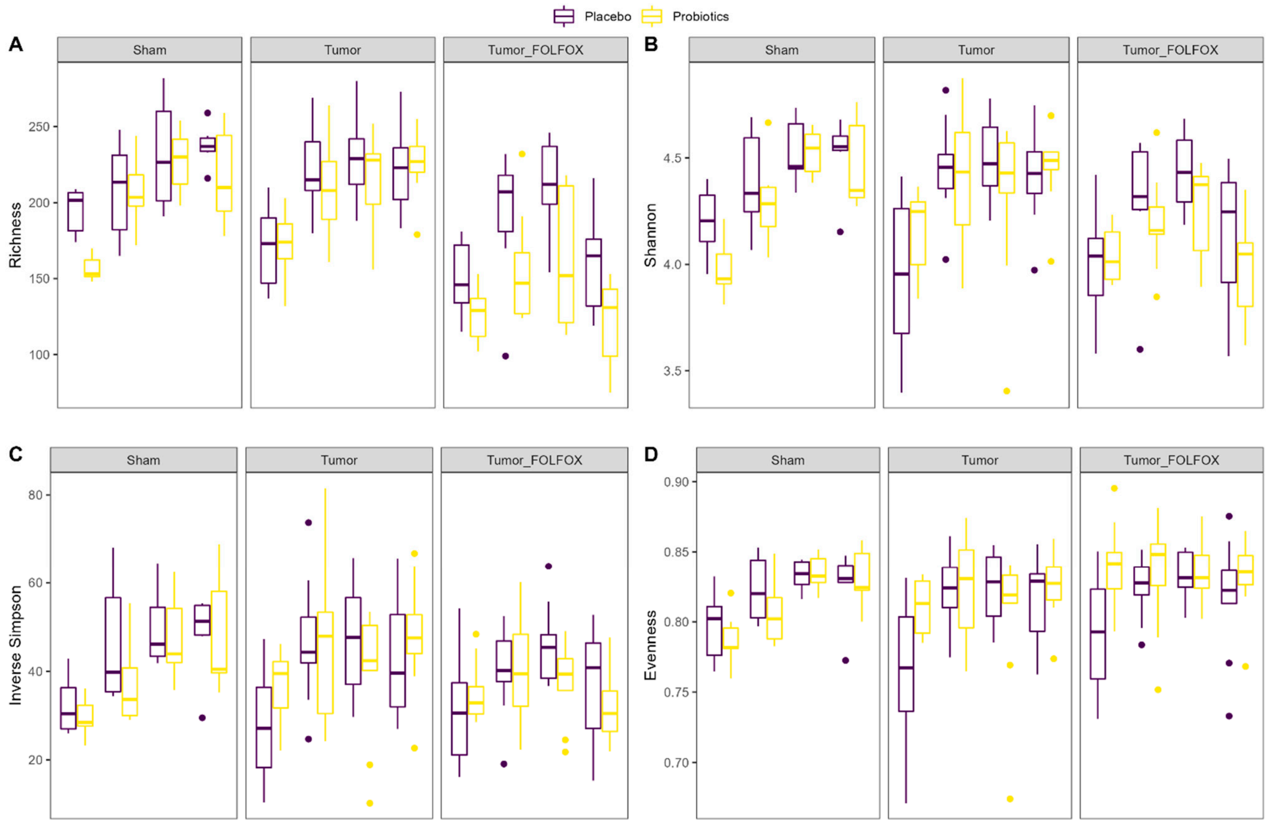

Figure S2. Alpha diversity panel. Calculated from non-normalized data.

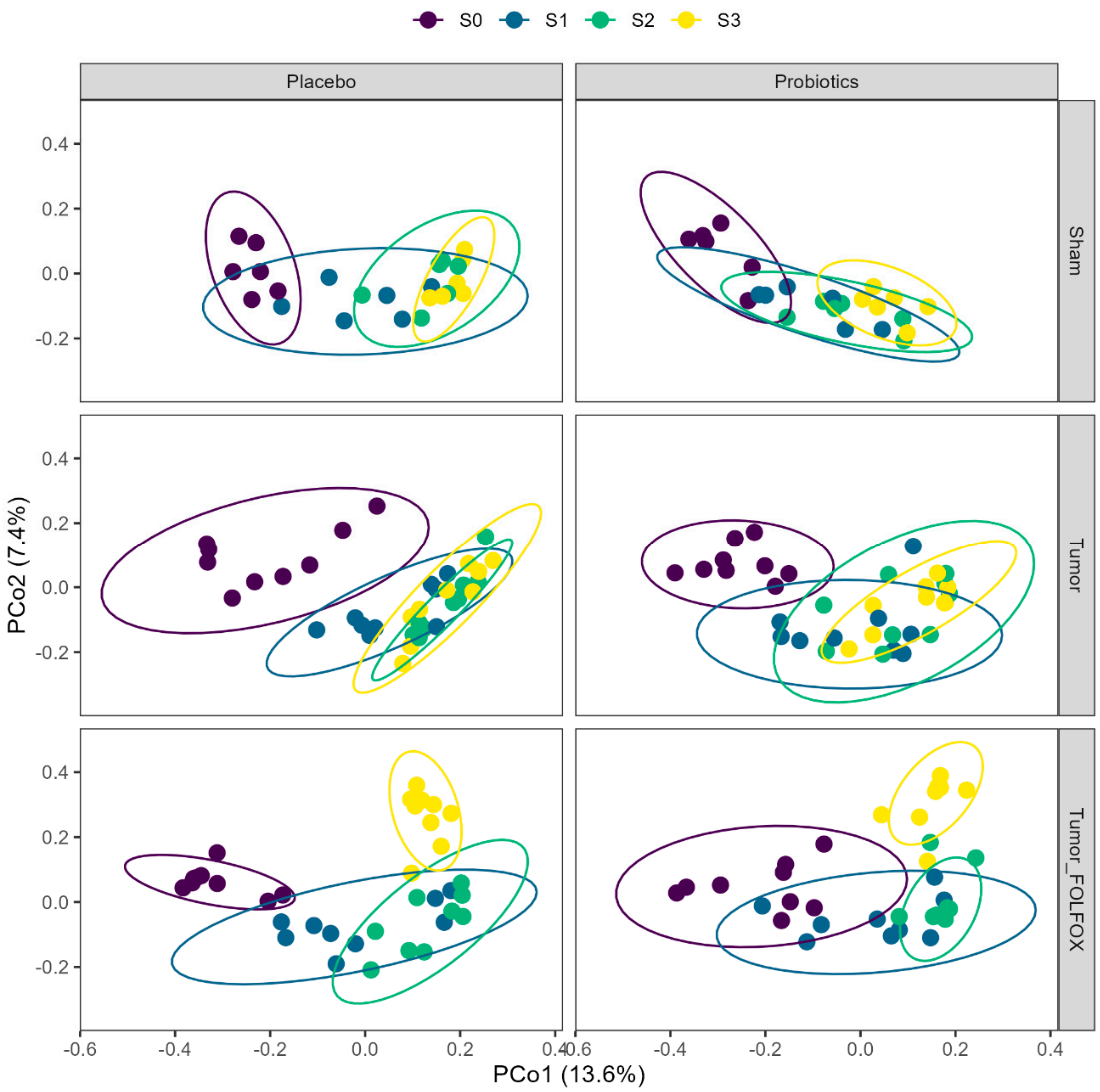

Figure S3. Principle coordinate analysis (PCoA) using Bray-Curtis dissimilarity.

Microbiome composition

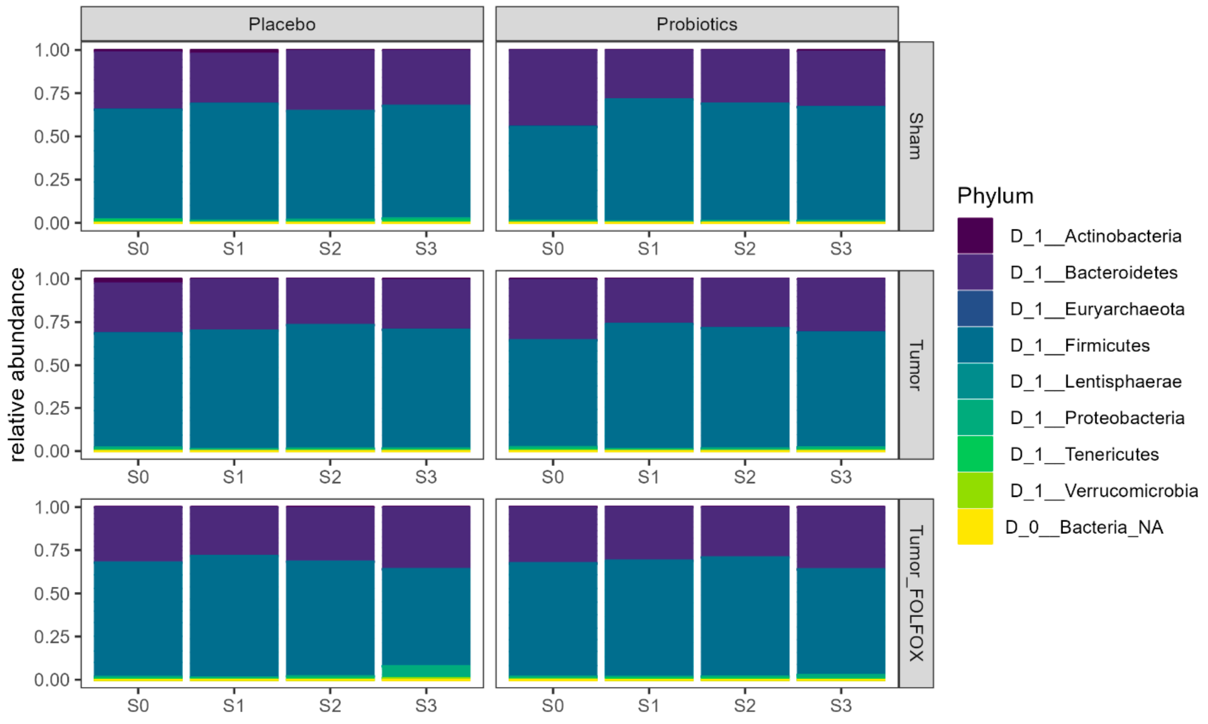

Figure S4. Phylum level microbiome composition

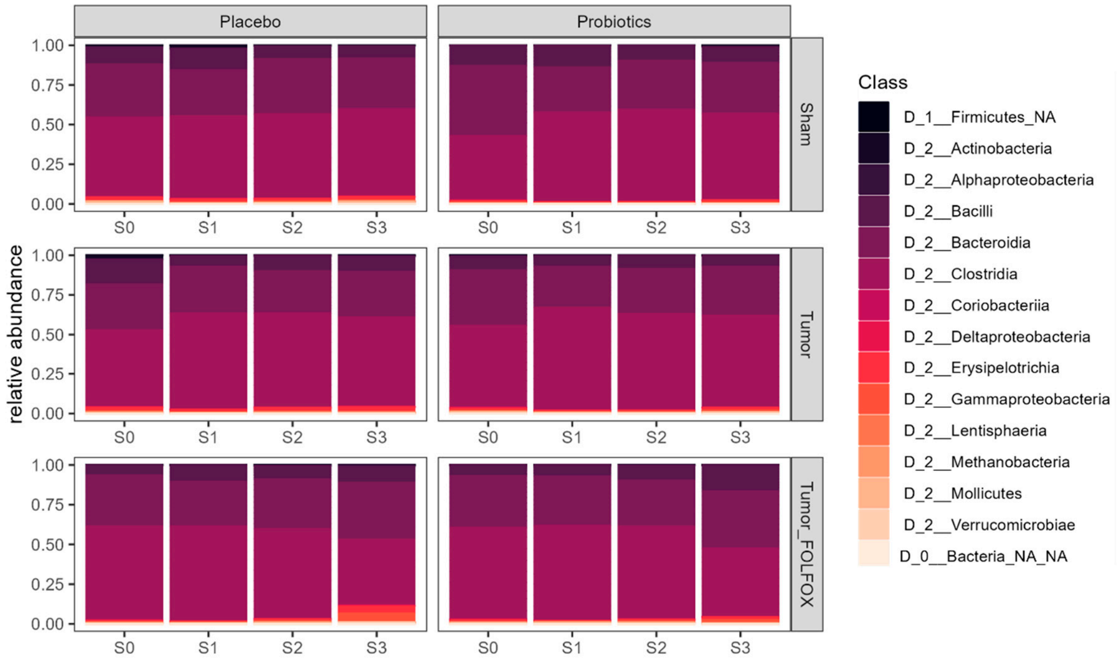

Figure S5. Class level microbiome composition

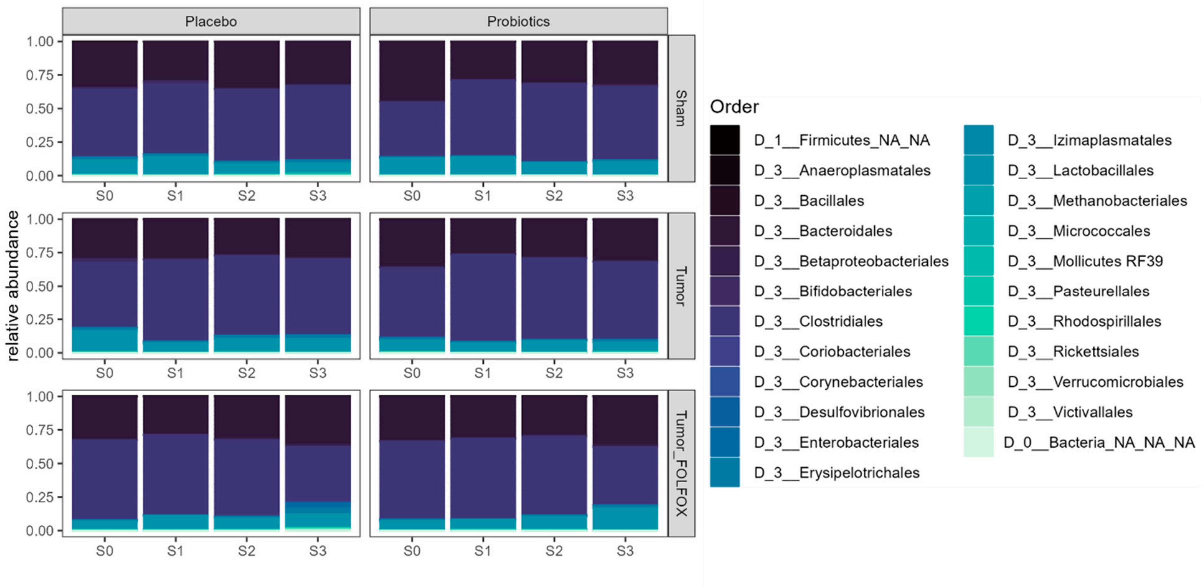

Figure S6. Order level microbiome composition

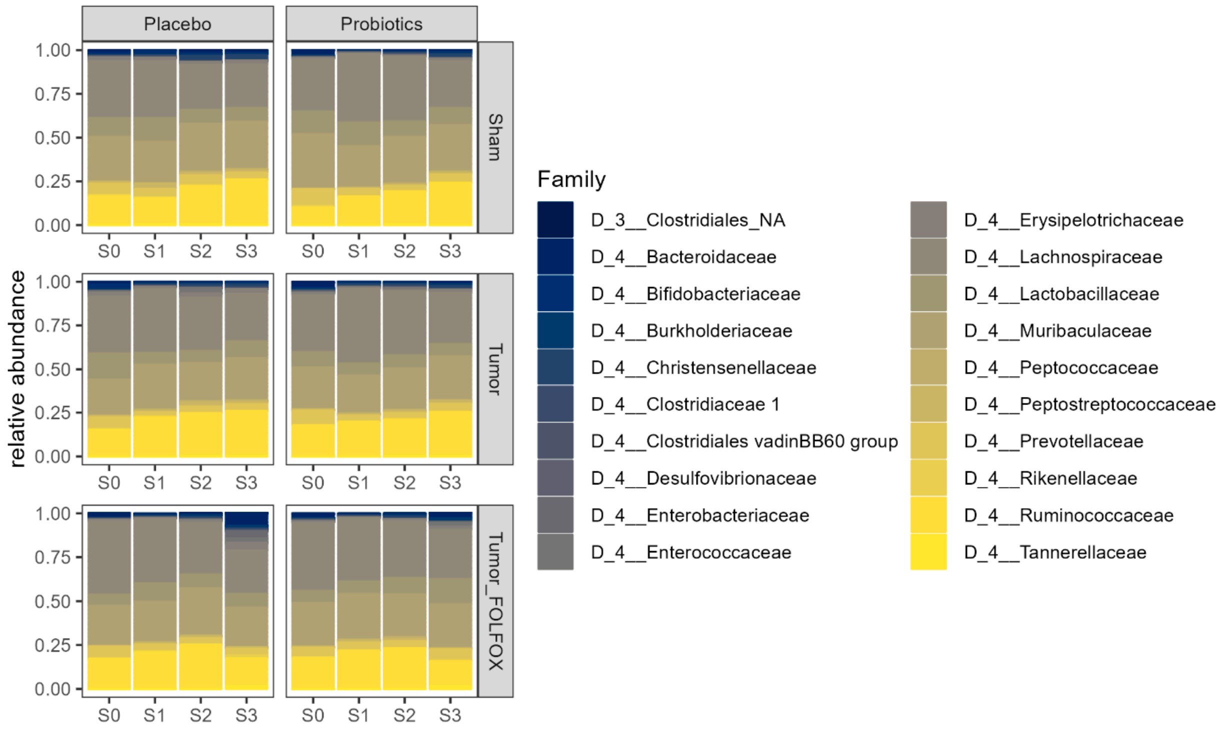

Figure S7. Family level microbiome composition

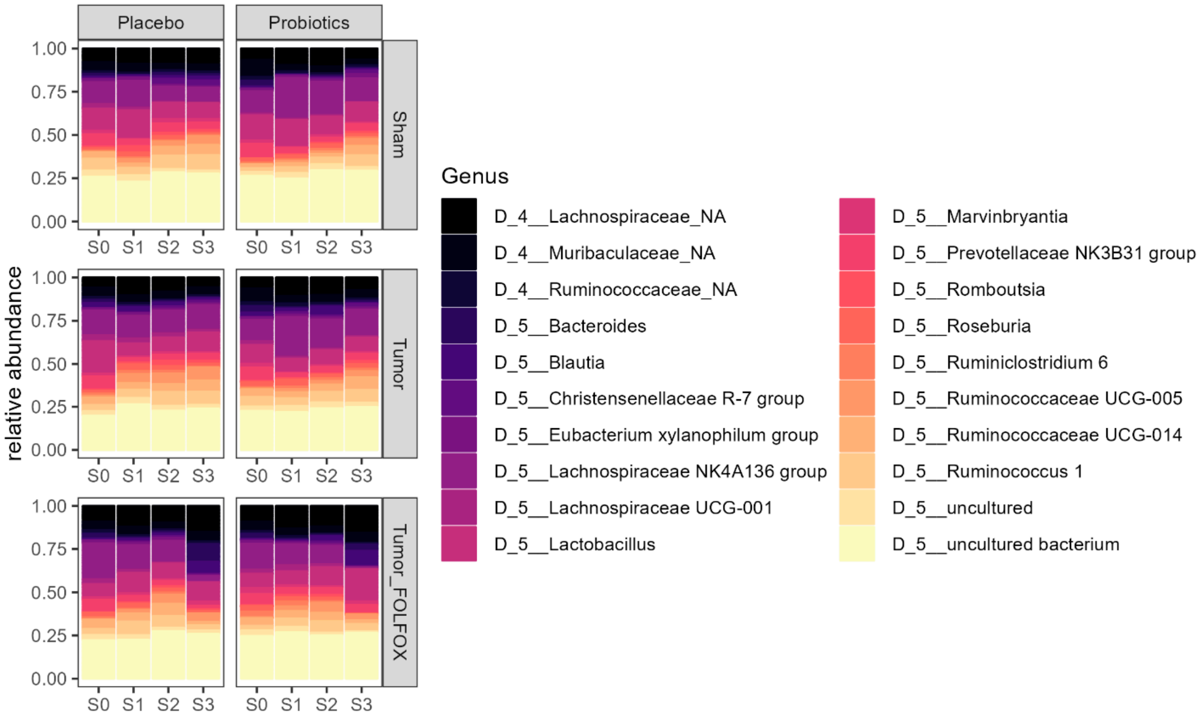

Figure S8. Genus level microbiome composition
